# When to use frovatriptan in migraine? A reply

**DOI:** 10.1007/s10194-011-0347-z

**Published:** 2011-05-04

**Authors:** Brigida Fierro, Stefano Omboni, Marco Bartolini, Lidia Savi, Lorenzo Pinessi

**Affiliations:** 1 Department of Neurology and Psychiatry, University of Palermo, Palermo, Italy; 2 Italian Institute of Telemedicine, Varese, Italy; 3 Department of Neuroscience, Polytechnic University of Marche, Ancona, Italy; 4 Department of Neurology, University of Torino, Turin, Italy

Dear Sir,

We read with interest the comments of Dr. Tfelt-Hansen [1] on the two recently published randomized controlled trials comparing patients’ preference (primary end-point) and efficacy (secondary end-points) of frovatriptan with respect to rizatriptan [2] and almotriptan [3]. In both studies, frovatriptan showed similar preference and short-term efficacy outcomes (pain relief and pain free episodes at 2 h) with respect to the other two triptans.

The principal concern of Dr. Tfelt-Hansen was the very early use of frovatriptan in these studies, making their results hardly comparable with those of previous randomized controlled trials [4–6], where patients waited until the headache was moderate or severe. In our studies [2, 3], patients were instructed to take one dose of study medication as early as possible after the onset of migraine and to treat at least 1 out of three attacks, but severity of headache at the time of drug intake was in the majority of patients moderate or severe. As a matter of fact, in the study by Savi et al. [2] 78% of attacks treated with frovatriptan and 74% of those treated with rizatriptan were of moderate or severe intensity. In the study by Bartolini et al. [3] 80% of overall treated attacks were of moderate or severe intensity and 97% of patients had at least one moderate or severe attack.

However, these studies had a cross-over design and thus the same patient was treated with both drugs in a randomized sequence. Moreover, early treatment in our studies better reflects reality compared to the artificial design of other studies where patients usually start treatment when the attack intensity becomes moderate or severe. Thus, the results of our studies are much more consistent than those of randomized controlled studies with a parallel group design.

In addition, the most significant result of our studies was a more sustained relieving effect on migraine symptoms with frovatriptan, with lower headache recurrence rates over the 48 h; these results are in line with previous reports comparing frovatriptan with placebo [7–9] or sumatriptan [10].
